# The need for coordination of research activities in pediatric lung diseases

**DOI:** 10.1186/s40348-016-0060-8

**Published:** 2016-07-27

**Authors:** Harald Ehrhardt, Klaus-Peter Zimmer

**Affiliations:** Department of General Pediatrics and Neonatology, Center for Pediatrics and Youth Medicine, Justus-Liebig-University, Universities of Giessen and Marburg Lung Center (UGMLC), Member of the German Lung Research Center (DZL), Feulgenstr. 12, D-35392 Gießen, Germany

During the recent years, the scientific knowledge of the pathomechanisms and new therapeutic options in pediatric lung diseases has dramatically increased which is reflected by the more and more increasing number of publications in this important field. Additionally, major scientific efforts have been bundled within national and international research networks like the German Center for Lung Research (DZL), the platforms of the European Respiratory Society (ERS), and the different Cooperation in Science and Technology (COST) actions [1, 2]. It was for the first time that the German Society for Pediatric and Adolescent Medicine (DGKJ) brought together international lung researchers in an English-speaking “DGKJ Scientific LUNG Symposium” at the annual meeting in Munich on September 3 and 4, 2016. The DGKJ invited frontiers in science covering important aspects of pediatric lung diseases to discuss their latest research findings with clinical experts in the field. The discussion of recent results from animal models and from molecular studies emphasized the need for an earlier and better exchange between basic research and clinical challenges of pediatrics to promote the translation of experimental results to patient care (and back) which was also recently promoted, i.e., by the scientific journal of the European Respiratory Society [3]. All together, the symposium demonstrated impressively the relevant similarities and overlaps of pathomechanisms between the broad spectrum of pediatric lung diseases which arouse from completely different origins including inborn, acquired, genetic, and environmental factors.

Pediatric lung disease already starts early in utero when the highly orchestrated process of lung development gets disturbed [4–7]. The balance of signaling pathways driving alveolar, mesenchymal, and vascular development including fibroblast growth factor 10 (FGF-10) signal transduction, the transforming growth factor beta (TGF-β), hypoxia-inducible factors (HIF), vascular endothelial growth factor (VEGF-A), and vitamin A-retinoid signaling represents the prerogative for undisturbed alveolo- and vasculogenesis. Surprisingly, not only the overstimulation of central growth signaling pathways including the nuclear factor “kappa-light-chain-enhancer” of activated B cells (NFkB) pathway but also the reduced baseline activity, i.e., in the absence of a central regulatory cytokine-like tumor necrosis factor alpha (TNF-α) can aggravate lung damage [4, 6, 7]. The complexity of regulation is further enhanced by the post-transcriptional control of protein production by microRNAs. Although many studies were able to identify potential candidates, the proof of causality is still mostly missing [5]. The central link between pulmonary inflammation and distortion of lung development is modulated by amniotic infection and a bundle of well-established clinical therapies including steroid exposure as well as the avoidance of mechanical ventilation and high oxygen exposure [6, 8]. Within the cell populations contributing to the development of chronic lung disease, mesenchymal stromal cells have attracted special attention during the recent years. Animal studies and a first human study showed a protective effect to the neonatal lung but a clear separation of the potential mechanisms of action including growth factor release, immunomodulation, and the substitution of injured cells is necessary before broad application within clinical trials [9, 10]. We are just beginning to understand the long-term consequences for these patients [6, 11]. During the presentations, it became clear that the major pediatric lung diseases like BPD, asthma, cystic fibrosis, and severe respiratory viral infections have common and distinct signaling pathways responsible for disease severity. Different genetic and epigenetic constellations, the exposure to certain environmental factors, and the heterogeneity of immune response determine the clinical phenotype of asthma, cystic fibrosis, and viral infections [12–17]. Even within a class of pathogens, the invader can take different endocytotic pathways and target different intracellular sites [16]. Despite the high complexity of immune reactions and pathomechanisms which might account for differences in phenotype and treatment responses, there are also promising results for potential candidates like granulocyte-macrophage colony-stimulating factor (GM-CSF) to treat different bacterial and viral infections with the identical targeted therapy [17].

To this end, the conclusions of the symposium pointed out that despite the heterogeneities between different pediatric lung diseases and even between different clinical phenotypes within one disease, the commonalities of major pathomechanisms highly encourage a comprehensive concept to elucidate the frontiers in science to the different pediatric lung diseases within a bundled interdisciplinary approach. Learning from the others’ cohorts and experimental results cannot only broaden the horizon but speed up the development and introduction of new and more specific therapies into the clinics to all children with pediatric lung diseases. We want to thank all speakers for their excellent presentations during the symposium and for their important contributions to this special edition.
